# Case Report: Olaparib Shows Satisfactory Clinical Outcomes Against Small Cell Esophageal Carcinoma With *ATM* Mutation

**DOI:** 10.3389/fonc.2022.808801

**Published:** 2022-04-11

**Authors:** Weiwei Wang, Xiaoyan Zhang, Yu Fang, Jia He, Jingjing Huang, Shanqing Li, Tonghui Ma, Li Li

**Affiliations:** ^1^ Department of Thoracic Surgery, Peking Union Medical College Hospital, Beijing, China; ^2^ Department of Translational Medicine, Genetron Health Technology, Co. Ltd., Beijing, China

**Keywords:** case report, olaparib, SCEC, ATM mutation, PARP inhibitor

## Abstract

Small cell esophageal carcinoma (SCEC) is a rare, undifferential type of cancer, with a high degree of malignancy and early systemic metastasis. Radio-chemotherapy and surgery have been used as the primary treatment strategies for SCEC, but they both result in poor prognosis. There is need to develop an optimal standard treatment for the disease to improve prognosis and limit the related mortality. In this study, we described identification of driver mutations in *ATM*, a gene involved in homologous recombination deficiency (HRD) pathway, using next-generation sequencing on primary lesion and peripheral blood of a SCEC patient, who experienced recurrence after resection and radio-chemotherapy. In addition, we subjected the patient to olaparib, a PARP inhibitor, for the treatment of tumor with HRD and obtained a partial response. This is the first evidence implicating olaparib in successful treatment of SCEC with *ATM* mutation. The findings suggest that targeting mutations in HRD genes using olaparib or actionable genetic mutations using corresponding drugs, may be an effective therapeutic option for SCEC, although this requires further investigation.

## Introduction

Small cell esophageal carcinoma (SCEC) is a rare and aggressive type of cancer with high relapse and mortality (1). SCEC is usually confirmed by histopathology, because X-ray images of patients diagnosed with this condition are similar to those of esophageal squamous cell carcinoma and adenocarcinoma. To date, limited treatment potions exist for SCEC, with most strategies employing radio-chemotherapy or surgery, which result in poor prognosis (2, 3). Overall, the optimum treatment approach for SCEC has not been developed. Given the rarity of SCEC, little is known regarding the potential role played by oncogenic factors in development of this cancer, with only one sample genomic data in Catalogue Of Somatic Mutations In Cancer (COSMIC; ID: COSS2080631) reported. However, rapid advances in genomics have led to discovery of drivers of pathogenic mutations in cancers, resulting in development of targeted drugs. For instance, olaparib, a targeted drug for PARP inhibitor (PARPi), has been used (alone or in combination with other drugs) for treatment of tumors with homologous recombination deficiency (HRD) (4). HRD pathways (KEGG hsa03440) include multiple genes, such as *ATM*, a gene for ataxia telangiectasia mutated protein (ATM) (5). A deficiency of this gene has been found to induce sensitivity to PARPi in other cancers (6–8) or esophagus cancer cell lines (9, 10). However, its role in treatment of esophagus cancer remains unknown. In the present study, we report identification of one driver mutation in the *ATM* gene, using a case of SCEC based on analysis of next generation sequencing data derived from an esophagus lesion and ctDNA (circulating tumor DNA). In addition, we show successful treatment of this condition using olaparib, with satisfactory clinical outcomes.

## Case Description

**Figure 1:**
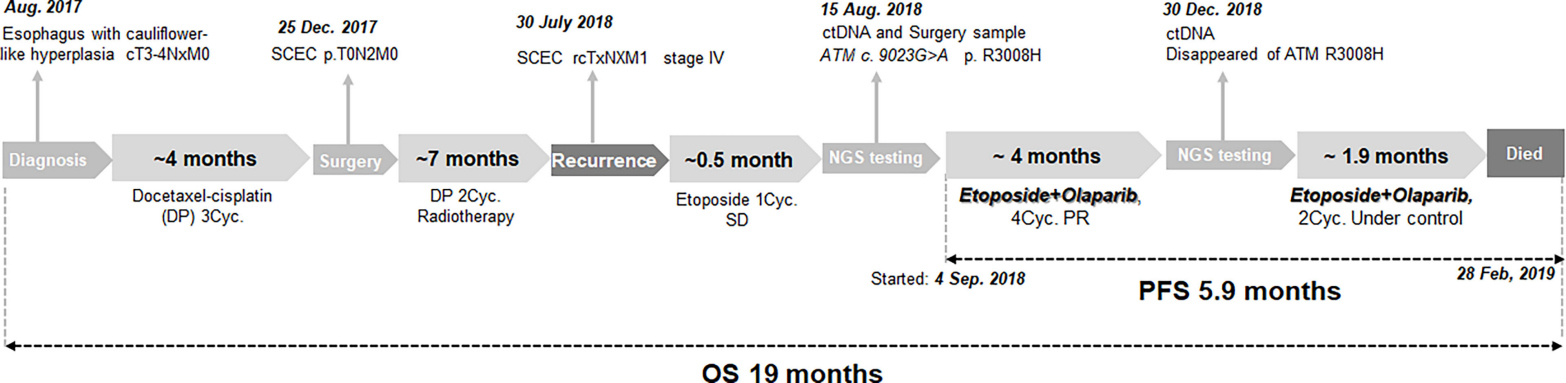
The flowchart of the case timeline to illustrate the diagnosis and treatment.

In August 2017, a man aged 56 years with smoking, drinking and family history of cancer, was presented to a local hospital with symptoms of dysphagia and stomach pains for nearly a month. A gastroscopy revealed cauliflower-like hyperplasia in the esophagus, about 21 to 26 cm from the incisors. A biopsy of this section at our hospital revealed tumor cells (cT3-4NxM0) (**Figure 1**). This patient was subsequently transferred to our hospital, he accepted 3 cycles of docetaxel-cisplatin (DP) regimen as neoadjuvant chemotherapy. CT showed the tumor reduction. He later underwent esophageal tumor resection and cervical lymph node dissection. Postoperative pathologic diagnosis revealed esophageal carcinoma, with lymph node metastasis (pT0N2M0, pIIIA). Immunohistochemical (IHC) staining of the resected tumor showed that AE1/AE3 and Ki-67 (index 80%) were partially positive, while CD56 (NK-1), CgA, Syn and TTF-1were positive. Based on these results, the patient was finally diagnosed with small cell esophageal carcinoma (SCEC), with lymph node metastasis. The patient was then treated with 2 cycles of DP as regimen as adjuvant chemotherapy, then subjected to adjuvant radiotherapy (DT50. 4Gy/28f, 1.8 Gy/f, 5f/w). The disease was well controlled.

**Figure 2:**
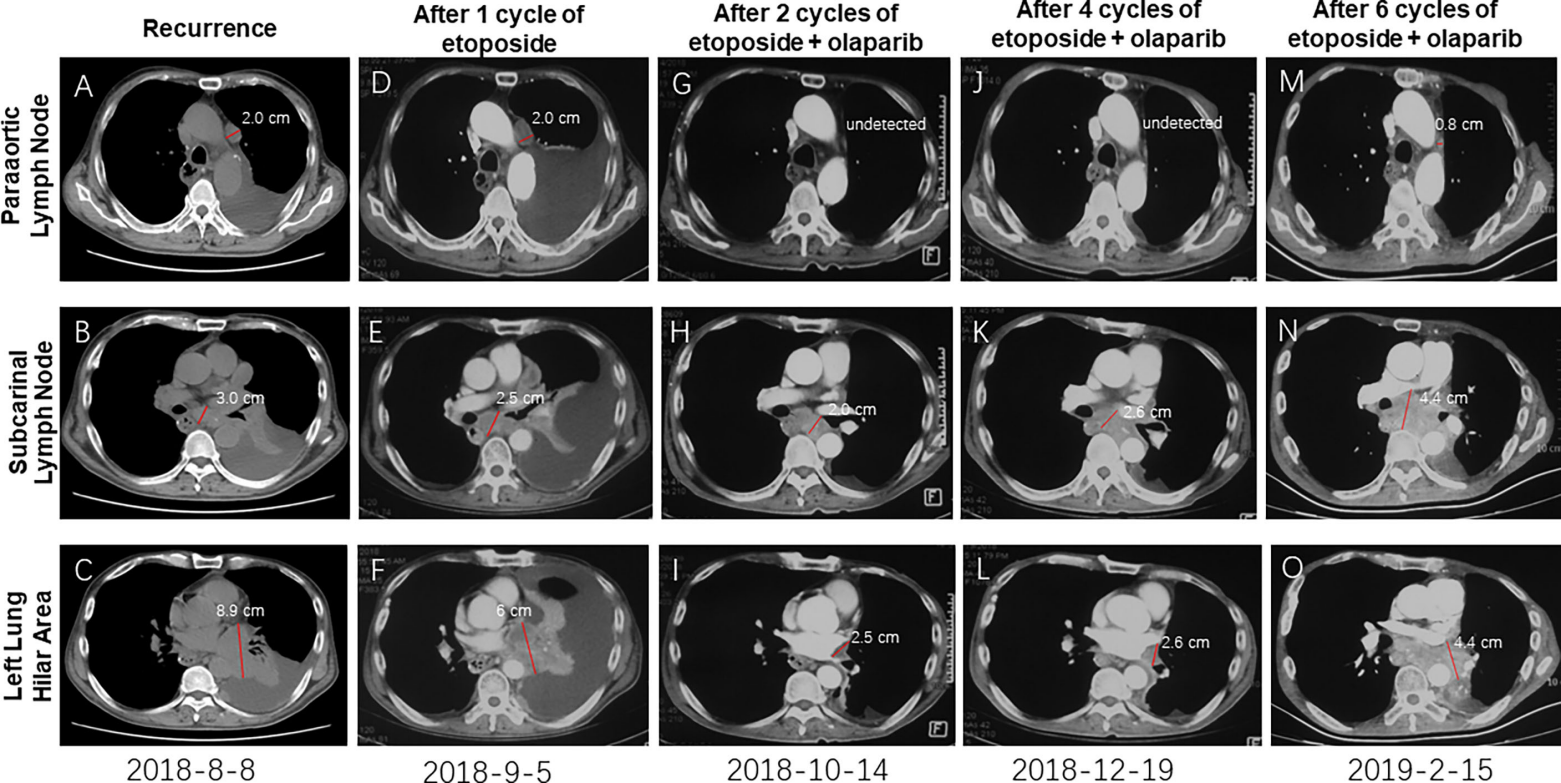
CT imaging showing the recurrent masses and the change of these masses after therapy. Masses after recurrence **(A–C)**; The recurrent masses kept stable after 1 cycle of etoposide **(D–F)**; The recurrent masses were obviously shrunk after 2 cycles of etoposide-olaparib **(G–I)**; These masses kept stable after 4 cycles **(J–L)** and 6 cycles **(M–O)** of etoposide-olaparib.

On 30^th^ July 2018, the patient went back to hospital complaining of breathlessness and fatigue. A CT scan revealed a 2.0cm mass in the paraaortic lymph node (**Figure 2A**), a 3.0 cm mass in the subcarinal lymph node (**Figure 2B**), and a large mass (8.9 cm) in the left lung hilar area (**Figure 2C**). Results from a pathological analysis of pleural effusion revealed SCEC cells (rcTxNxM1, stage IV). The patient was subjected to chemotherapy with etoposide (50mg, qd, d1-d10, q3w). After 1 cycle, CT showed similar size masses as before, the efficacy was stable (**Figures 2D–F** vs. **Figures 2A–C**). Results from analysis of hybridization-based targeted next generation sequencing of ctDNA by a 180-gene panel (OncoFocus, Genetronhealth) and resected tumor by a 831-gene panel (OncoPanscan panel,Genetronhealth) which cover revealed the same mutation (c. 9023G>A, p. R3008H) in the *ATM* gene. Olaparib (200mg, bid) was then added to the treatment on 4^th^ September 2018. After 2 cycle of olaparib plus etoposide, CT scans showed marked shrinking of the masses (**Figures 2G–I**), the efficacy reached to partial response. Therefore, the patient continued using this regimen. A CT scan performed on 19^th^ December 2018, after 4 cycles of treatment, revealed that the masses were well controlled (**Figures 2J–L**). NGS (OncoFocus, Genetronhealth) performed on the ctDNA taken at this time revealed that the mutation (R3008H) in the *ATM* gene had disappeared. The patient continued to use this regimen, with further CT scans (on 15^th^ February 2019) showing that the masses were still under control (**Figures 2M–O**). However, the patient died of respiratory failure and coagulation problem half a month later. The PFS of olaparib plus etoposide was 5.9 months, the overall survival was 19 months (**Figure 1**).

## Discussion

Small cell esophageal carcinoma, which is similar to small cell carcinoma of lungs, but not other esophageal cancers, accounts for only 0.4-2.8% of all primary esophageal carcinoma cases (3). In recent years, although increasing studies have reported SCEC, most of them have focused on retrospectively analyzing multimodal therapeutic approaches that combine surgery, radiotherapy and chemotherapy (3, 11). Current therapies for SCEC have always resulted in poor prognosis (3, 11). Consequently, there is no optimal standard therapeutic approach for treating SCEC. In the present study, a *ATM* mutation were identified in a 56 years-old man diagnosed with SCEC. The patient showed partial response after the treatment of olaparib. Overall, we provided evidence of a novel potential therapeutic option for SCEC patients with *ATM* gene mutation.

**Figure 3:**
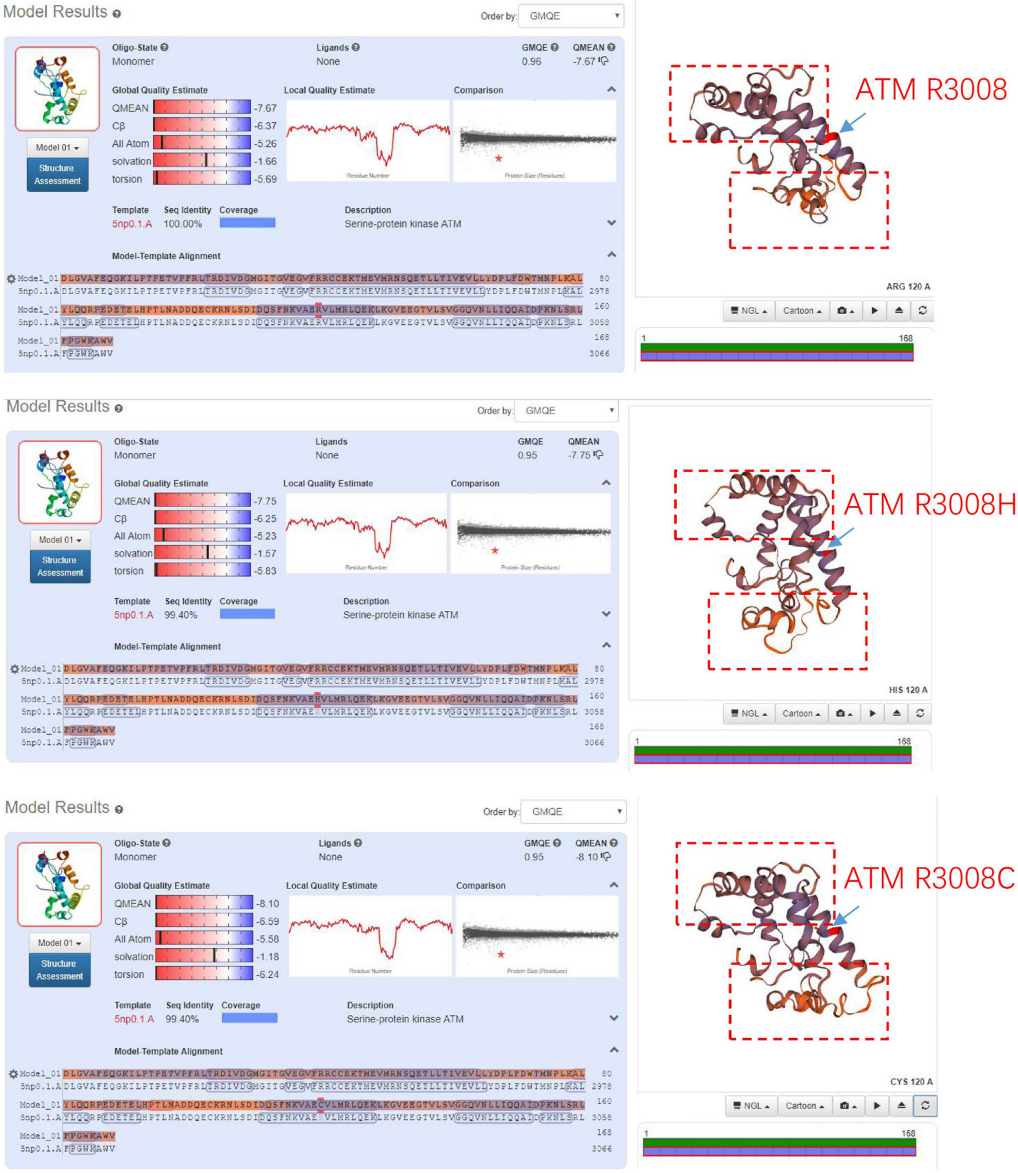
Comparing the tertiary structures of the activation loop and FATC domain of *ATM* gene that containing R3008H or R3008C mutant.

*ATM* is one homologous recombination gene that regulates a variety of downstream proteins, such as p53, BRCA1, checkpoint kinase CHK2 and DNA repair protein NBS1 (5). In addition, it plays a central role in the activation of DNA damage responses following double-strand breaks (DSB) (7). Somatic mutations in the *ATM* gene have been found in various tumors (12), such as lung adenocarcinoma (13), pancreatic ductal adenocarcinoma (14), metastatic prostate cancer (6) and gastric cancer (7). ATM R3008C/H/L were found to be recurrent mutations in TCGA data. Comparing with ATM R3008C mutation, which is well-known as oncogenic mutation, ATM R3008H mutation has not been functionally or clinically validated. Analysis of tertiary structures of the activation loop and FATC domain of the *ATM* gene, that included R3008 mutant, showed that R3008H mutation had similar structural changes like R3008C (**Figure 3**), suggesting the R300H mutation tend to be a driver of mutations.

PARP family of enzymes has been implicated in DNA repair mechanism, by indirectly helping in repair of DSB following ATM activation. PARPi-derived compounds, such as olaparib, rucatinib, niraparib and talazoparib, have been found to interfere with the repair functions of PARP enzymes by competitively binding onto their NAD binding sites (7). Moreover, ATM deficiency has been observed to improve sensitivity to PARPi, olaparib, in gastric cancer (7), prostate cancer (6), and genitourinary malignancies (8). In the present study, incorporation of olaparib to the treatment of the patient who had *ATM* mutations resulted in 5.9-month progression-free survival (**Figure 1**). This is the first report of the use of olaparib in successful treatment of SCEC patient with *ATM* mutation.

## Conclusions

Since SCEC is a rare malignancy without standard treatment, we hope that the treatment based on the diagnoses results of actionable genetic mutations could potentially generate new therapeutic options against this disease. We identified an *ATM* gene mutation in a patient diagnosed with SCEC recurrence and achieved partial response after olaparib treatment. To our knowledge, this is the first report of a targeted therapy applied for treatment of SCEC, based on information of genetic alterations generated by next-generation sequencing. This is also the first report showing successful management of SCEC with *ATM* mutation using olaparib. However, the results herein were only derived from one patient, therefore further investigations are needed to validate the findings.

## Data Availability Statement

The original contributions presented in the study are included in the article. Further inquiries can be directed to the corresponding authors.

## Ethics Statement

Ethical approval was not provided for this study on human participants because we have obtained an informed consent from the relative of the patient. The patients/participants provided their written informed consent to participate in this study.

## Author Contributions

LL and TM contributed to conception and design of the study. JJH and YF prepared figures and background research. WW and XZ wrote the first draft of the manuscript. JH and SL wrote sections of the manuscript. All authors contributed to the article and approved the submitted version.

## Conflict of Interest

XZ, YF, JJH, and TM have disclosed that they are employees of Genetron Health.

The remaining authors declare that the research was conducted in the absence of any commercial or financial relationships that could be construed as a potential conflict of interest.

## Publisher’s Note

All claims expressed in this article are solely those of the authors and do not necessarily represent those of their affiliated organizations, or those of the publisher, the editors and the reviewers. Any product that may be evaluated in this article, or claim that may be made by its manufacturer, is not guaranteed or endorsed by the publisher.
